# Endoscopic full-thickness resection of gastric and duodenal subepithelial lesions using a new, flat-based over-the-scope clip

**DOI:** 10.1007/s00464-017-5989-8

**Published:** 2017-12-27

**Authors:** Wouter F. W. Kappelle, Yara Backes, Gerlof D. Valk, Leon M. G. Moons, Frank P. Vleggaar

**Affiliations:** 1 Department of Gastroenterology and Hepatology, University Medical Center Utrecht, P. O. Box 85500, 3508 GA Utrecht, The Netherlands; 2 Department of Endocrine Oncology, University Medical Center Utrecht, Utrecht, The Netherlands

**Keywords:** Over-the-scope clips, Endoscopy, Full-thickness resection, Subepithelial tumor

## Abstract

**Background:**

Surgical resection of upper gastrointestinal (GI) subepithelial tumors (SETs) is associated with significant morbidity and mortality. A new over-the-scope (OTS) clip can be used for endoscopic full-thickness resection (eFTR). We aimed to prospectively evaluate feasibility and safety of upper GI eFTR with a new, flat-based OTS clip.

**Methods:**

Consecutive patients with a gastric or duodenal SET < 20 mm were prospectively included. After identification of the lesion, the clip was placed and lesions were resected. Patients were followed for 1 month to assess severe adverse events (SAEs); 3–6 months after eFTR, endoscopy was performed.

**Results:**

eFTR was performed on 13 lesions in 12 patients: 7 gastric and 6 duodenal SETs. Technical success was achieved in 11 cases (85%). In all 11 cases, R0-resection was achieved. In all 6 duodenal cases and in one gastric case, FTR was achieved (64%). One SAE (pain) was observed after eFTR of a gastric SET. After eFTR of duodenal SETs, several SAEs were observed: perforation (*n* = 1), microperforation (*n* = 3), and hemorrhage (*n* = 1). During follow-up endoscopy, the clip was no longer in situ in most patients (7 of 10; 70%).

**Conclusions:**

eFTR with this new flat-based OTS clip is feasible and effective. Although gastric eFTR was safe, eFTR in the duodenum was complicated by (micro)perforation in several patients. Therefore, the design of the clip or the technique of resection needs further refinement to improve safety of resection of SET in thin-walled areas such as the duodenum before being applied in clinical practice.

Dutch trial register: NTR5023.

**Electronic supplementary material:**

The online version of this article (10.1007/s00464-017-5989-8) contains supplementary material, which is available to authorized users.

Subepithelial tumors (SETs) located in the stomach or duodenum, such as neuroendocrine tumors (NETs) and gastrointestinal stromal tumors (GISTs), can cause significant morbidity and, in some cases, mortality. If necessary, SETs are usually resected by laparoscopy. However, laparoscopic resection of such tumors, especially in the duodenum, is associated with considerable morbidity [1–4]. Endoscopic removal is advised for small duodenal SETs (< 1 cm) and surveillance or endoscopic removal (depending on suspected etiology of the lesion) for gastric SETs < 1 cm [5]. The guideline advices surgical resection for lesions > 2 cm and careful consideration for SETs 1–2 cm in size. However, small SETs are often treated conservatively since viable treatment options are lacking; this approach is safer, but may leave the patient with bothersome symptoms, uncertainty on the diagnosis, and regular surveillance endoscopies. Should endoscopic removal be attempted, endoscopic mucosal resection (EMR) can be performed if the tumor is smaller than 1–1.5 cm. However, EMR is associated with a low rate of R0-resection and a high complication rate [6]. Another option is endoscopic mucosal dissection (ESD). The R0-resection rate of ESD is higher compared with EMR but the risk of perforation is higher [7, 8]. In the duodenum, EMR is preferred over ESD, since the risk of perforation is > 30% with ESD [9].

This problem of low R0-resections may be overcome with a full-thickness resection which includes the muscularis propria in the specimen such as in a laparoscopic wedge excision. Recently, endoscopic full-thickness resection (eFTR) using over-the-scope (OTS) clips has gained interest as an alternative to laparoscopic surgery and EMR/ESD in this patient category. Despite the limited invasiveness of the procedure, eFTR is a potentially curative treatment and allows for a definitive histopathological diagnosis, which is often difficult in smaller SETs. A new OTS clip with a flat base (Padlock Pro-select, Aponos Medical Corp., Kingston, NH, USA) has recently become available [10]. The flat base of the clip is thought to facilitate snaring of the tumors after deployment of the clip, thus increasing the chance of achieving R0-resection. Successful eFTR with this new flat-based OTS clip in gastric or duodenal SETs has been reported in only one case thus far [11]. Herein, we report our experience with eFTR with this new flat-based OTS clip in gastric and duodenal SETs.

## Patients and methods

Between January 2015 and July 2016, patients were prospectively included in this study at the Department of Gastroenterology and Hepatology at the University Medical Center Utrecht, The Netherlands. Patients were eligible for inclusion if they were ≥ 18 years of age, if previous endoscopic ultrasound showed a submucosal lesion ≤ 20 mm in the gastric or duodenal wall, and if resection was indicated. Indications for eFTR included (suspected) hormone-producing NET or (in)definite histology. In case of indefinite histology, the aims of eFTR were to remove uncertainty of the patient with regard to unknown etiology of the lesion, to stop potentially unnecessary surveillance, and to remove the risk of metastasis [12]. The research protocol was approved by the institutional review board. Patients provided informed consent for endoscopy and data collection.

### The over-the-scope clip

**Fig. 1:**
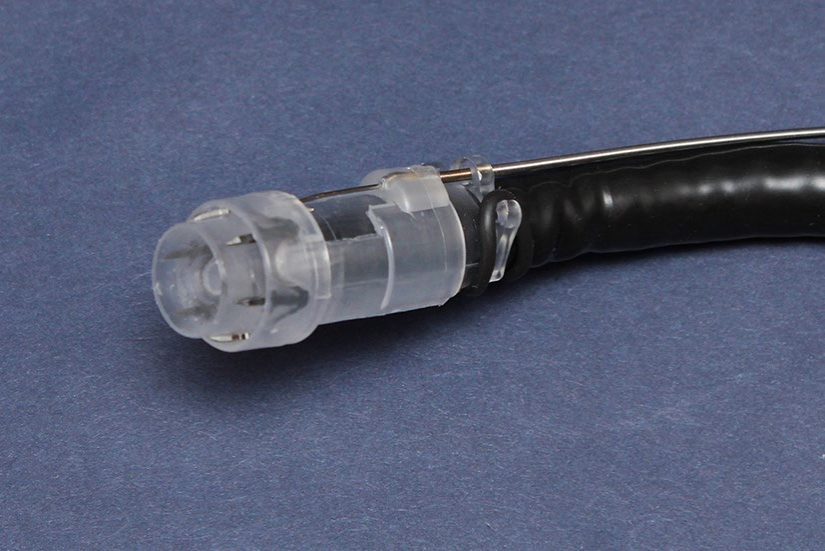
The Padlock clip, preassembled on applicator cap, with the external deployment mechanism alongside the endoscope, mounted on the tip of an endoscope

**Fig. 2:**
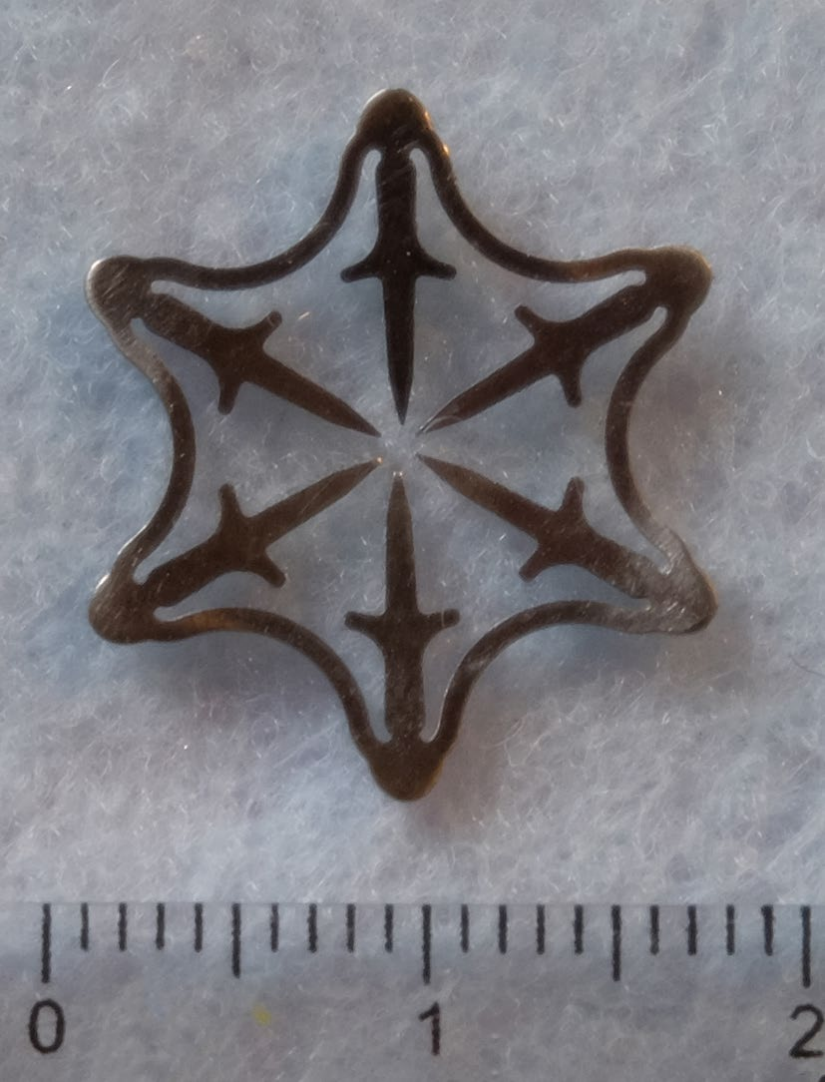
The Padlock clip has a hexagonal shape and is flat based once deployed

The Padlock system consists of a clip, a cap, and a deploying mechanism (Fig. 1). The clip consists of a nitinol ring with six “swords” of 7 mm, which is preassembled on a transparent applicator cap with a chamber of 11.5–14 millimeter (depending on the endoscope used). The swords point forward when the clip is situated on the cap (Fig. 1). The swords turn inward when the clip is deployed, thereby puncturing, lifting, and approximating the targeted tissue (Fig. 2). Once deployed, the clip is flat based and has a hexagonal shape. The applicator cap is mounted on a regular or therapeutic endoscope; the clip is deployed by a thumb press mechanism. The trigger wire is located alongside the endoscope.

### Endoscopic full-thickness resection

Procedures were performed by two experienced endoscopists (FV, LM) with a GIF-HQ190, GIF-1TH190, or GIF-2TH180 endoscope (Olympus, Hamburg, Germany). Patients were placed in the left lateral decubitus position and vital functions were monitored during the procedure. Patients were sedated using monitored anesthesia care with propofol. All lesions were visualized prior to resection with esophagogastroduodenoscopy (EGD) and endoscopic ultrasound (EUS) to confirm that the size of the lesion was appropriate and that the lesion was indeed located in the submucosa.

**Fig. 3:**
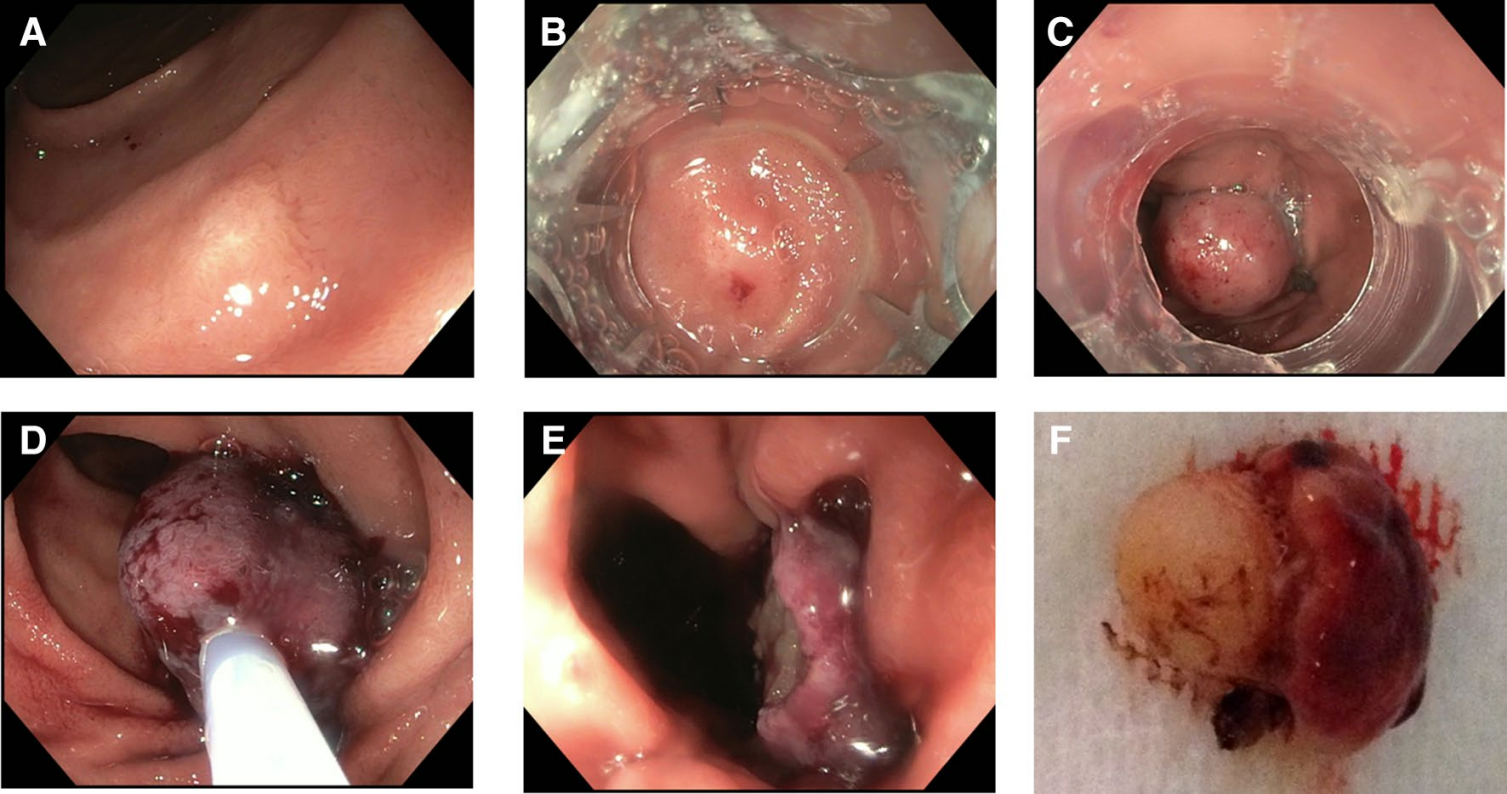
After identification of the duodenal lesion (**A**), the cap was placed on the endoscope and introduced (**B**). Thereafter, the clip was deployed, thereby creating a pseudopolyp (**C**). Hereafter, an electrosurgical snare was placed around the pseudopolyp (**D**) and the lesion was removed in auto-cut mode (**E**). The resected specimen (**F**) was sent for pathological evaluation

After identification of the target lesion (Fig. 3A), the endoscope was removed. After assembly of the cap, the scope with the cap was re-introduced (Fig. 3B) and the lesion was suctioned into the applicator cap, after which the clip was deployed. This way, a pseudopolyp was created with the target lesion situated above the deployed clip (Fig. 3C). Hereafter, the endoscope was removed and the cap was removed from the endoscope. After reintroduction of the endoscope, a 20-mm electrosurgical snare (Boston Scientific, Natick, MA, USA) was advanced through the working channel. The snare was placed around the pseudopolyp under close endoscopic visualization to ensure that the snare was not placed around the clip (Fig. 3D). Subsequently, the lesion was removed en bloc with the snare in auto-cut mode (Fig. 3E, Supplemental video 1). The resected specimen was retrieved with the snare (Fig. 3F). The base of the pseudopolyp was inspected for evidence of hemorrhage or perforation thereafter. The resected specimen was pinned on a paraffin block and was sent to the pathologist for histopathological assessment. Additionally, size, layer of origin, and margins of the tumor were evaluated.

### Endpoints

We aimed to assess technical feasibility, R0-resection rate, full-thickness resection rate, and safety. Technical success was defined as successful placement of the clip and removal of the lesion, without macroscopic remnant tissue left in situ. R0-resection was defined as microscopic SET-free horizontal and vertical margins in the specimen. Full-thickness resection was defined as the inclusion of muscularis propria in the specimen. Complications were graded according to the Clavien–Dindo classification: Grade I, no treatment or only certain medications required; Grade II, conservative management (including antibiotics); Grade III, surgical/endoscopic/radiologic intervention; Grade IV, ICU management; Grade V, death [13, 14].

### Follow-up

After eFTR, patients were hospitalized for one night. Prophylactic proton pump inhibitors were prescribed in all patients. The first 6 h after the procedure only clear liquids were given. If signs of infection or severe pain was observed, admission was prolonged at the discretion of the treating physician. Follow-up consisted of telephone calls after 1 week and 1 month to inquire about adverse events. Follow-up endoscopy was performed after 3–6 months.

### Statistical analysis

Categorical variables were expressed as proportions; continuous variables were expressed as medians (interquartile range; IQR) or means (standard deviation; SD), where appropriate. Since this was a feasibility study, no power analysis to calculate sample size was performed. The analyses were performed using SPSS version 23 (SPSS Inc., Chicago, Illinois, USA), and *p* values < 0.05 were considered statistically significant.

## Results

A total of 12 patients were included [male *N* = 8 (67%), mean age 52.8 ± 12.1 years] in whom 13 SETs were removed with eFTR. In one patient, two SETs were removed on separate occasions. Indications for eFTR included NET (*N* = 4), GIST (*N* = 2), inability to obtain histology (*N* = 1), and indefinite histopathology of biopsies (*N* = 6) obtained during earlier EGD and EUS. Six eFTRs in five patients were performed in the duodenum (all proximal to ampulla of Vater) and seven eFTRs were performed for gastric SETs. Mean size of the lesions was 11 ± 4 mm. Baseline characteristics of the patients and lesions and the results of eFTR are shown in Table 1.

**Table 1:** Lesion characteristics and resection results

Pt no	Gender	Age	Location	Layer	Indication	Lesion size, mm	Success	R0	FTR	Specimen size, mm	Pathology	SAE	Hospital admission, days
			Duodenum										
1	Male	51.7	Bulb	4	No histology	13.0 × 3.3	Yes	Yes	Yes	23 × 24 × 8	Brunneroma	No	2
2	Male	44.1	Descending part	3	NET	4.0 × 4.0	Yes	Yes	Yes	13 × 10 × 5	Gastrinoma	Microperforation	4
3	Male	60.7	Descending part	4	Pathology Inconclusive	10.0 × 10.0	Yes	Yes	Yes	15 × 15 × 6	Ectopic pancreas	Hemorrhage	10
4	Male	44.4	Descending part	3	NET	9.0 × 9.0	Yes	Yes	Yes	15 × 12 × 9	Gastrinoma	Microperforation	7
4	Male	40.7	Descending part	3	NET	10.0 × 10.0	Yes	Yes	Yes	24 × 17 × 3	Gastrinoma	Perforation	14
5	Female	61.9	Descending part	3	NET	5.0 × 5.0	Yes	Lost	Lost	Lost	Gastrinoma	Microperforation	7

			Stomach										
6	Female	54.1	Lesser curve	4	GIST	13.0 × 8.0	Yes	Yes	No	18 × 15 × 9	GIST	No	1
7	Male	69.7	Greater curve	4	GIST	18.0 × 16.0	No	NA	NA	NA	NA	No	NA
8	Male	69.4	Lesser curve	2	Pathology Inconclusive	13.0 × 6.0	Yes	Yes	Yes	Unknown	Duplication cyst with ectopic pancreas	No	1
9	Female	46.6	Fundus	4	Pathology Inconclusive	20.0 × 15.0	No	NA	NA	NA	NA	No	NA
10	Male	38.6	Antrum	4	Pathology Inconclusive	10.0 × 10.0	Yes	Yes	No	15 × 9 × 3	Ectopic pancreas	No	1
11	Female	62.2	Antrum	2	Pathology Inconclusive	12.0 × 8.0	Yes	Yes	No	13 × 10 × 7	Reactive gastric epithelium	No	1
12	Male	34.2	Antrum	2	Pathology Inconclusive	9.7 × 3.5	Yes	Yes	No	12 × 12 × 4	Inflammatory fibroid polyp	Pain	2

*FTR* full-thickness resection, *SAE* severe adverse event, *GIST* gastrointestinal stromal tumor, *NET* neuroendocrine tumor, *layer 2* muscularis mucosae, *layer 3* submucosa, *layer 4* muscularis propria

### Procedure outcomes

Technical success was achieved in 11 (85%) cases (Table 1). In two gastric lesions, suction of SETs in the cap was unsuccessful. These lesions measured 18 and 20 mm and were located in the fourth layer on EUS (muscularis propria). In one case, the cap could not be advanced beyond the pharynx due to edema caused by manipulation with the endoscope in the pharynx with the device in place. Six months later, endoscopy was repeated and clip placement was successful this time. In general, gastric lesions were found to be more difficult to place into the cap than duodenal lesions. In all successful cases, difficulty of the entire procedure was considered easy by the endoscopists. In one gastric eFTR, the SET was not situated within the pseudopolyp after clip placement. The clip was removed with a rat tooth forceps and a second clip was successfully placed. In another gastric eFTR, there was doubt whether the SET was captured in the pseudopolyp. Radial EUS (GF-UE160-AL5, Olympus, Hamburg, Germany) confirmed this and the SET was resected without difficulties. Mean total procedure time was 35 (± 10) min. Mean procedure time from the moment of insertion of the clip until removal of the endoscope was 18 (± 8) min. All successfully removed lesions were resected en bloc. Follow-up endoscopy was available in 10 of 11 successfully treated cases and revealed no local recurrences. In three cases, the clip was still in situ.

### Pathology results

Sizes of the resection specimens are shown in Table 1. In the duodenum, histopathology revealed brunneroma (*n* = 1), ectopic pancreas (*n* = 1), and gastrinoma (*n* = 4). Gastric lesions were GIST (*n* = 1), duplication cyst with ectopic pancreas (*n* = 1), ectopic pancreas (*n* = 1), reactive gastric epithelium (*n* = 1), and inflammatory fibroid polyp (*n* = 1). Interestingly, R0-resection was achieved in all cases. Full-thickness resection was achieved in all duodenal cases (*n* = 6) and in one of five gastric cases.

### Safety

Five of six duodenal eFTRs were complicated by immediate adverse events (within 10 h). One case was complicated by post-procedural hemorrhage and repeat endoscopy was performed and hemostasis was achieved with coagulation forceps; further treatment consisted of blood transfusion and coiling of the bleeding vessel (Grade III). Three patients reported having severe pain; on abdominal CT scan, fat stranding but no signs of perforation were observed. All three patients were diagnosed with a microperforation and were treated with opiates and antibiotics (Grade II). Finally, one patient was diagnosed with a perforation on abdominal CT scan which was treated conservatively with opiates and antibiotics (Grade II). Despite adjustments in technique (i.e., leaving more tissue in situ on top of the clip), adverse events occurred and the inclusion of duodenal cases was halted. Median post-procedural in-hospital stay was 2.0 days (IQR 1.0–7.0).

After gastric eFTR (*n* = 7), one patient reported pain with no signs of perforation on abdominal CT; treatment consisted of opiates (Grade I). No other severe adverse events (SAEs) were observed. The patient in whom the procedure was ceased due to esophageal edema reported dyspnea after the failed eFTR. Saturation was > 95%, infection parameters were low, and no symptoms of perforation or pneumonia were observed. The dyspnea resolved within hours without intervention (Grade I). Median post-procedural hospital stay was 1.0 day (IQR 1.0–1.5).

## Discussion

This is the first prospective study on eFTR of gastric and duodenal SETs. The current study showed that eFTR is technically feasible in both locations with the new flat-based Padlock OTS clip. Interestingly, R0-resection was achieved in all technically successful cases. Therefore, the utilized suction-and-entrap method seems correct when using this clip. Unfortunately, eFTR in the duodenum was complicated by (micro)perforation in several patients. The current technique of eFTR or the design of the clip therefore needs to be optimized for eFTR in the duodenum before application in clinical practice can be advised. In gastric eFTR, on the other hand, a favorable safety profile was observed which, combined with an optimal R0-resection rate, makes eFTR with this clip an interesting new option for endoscopic resection of gastric SETs.

Although alternative resection techniques (i.e., EMR, ESD, and surgery) are available for SETs < 20 mm, these techniques have important limitations. The duodenum is a notoriously difficult location for (endoscopic) resection of SETs. EMR is associated with a relatively low rate of R0-resection, uncertainty of complete resection if piecemeal resection is performed, and a high complication rate; in a recent report on sporadic neuroendocrine tumors in the duodenum, certain R0-resection was achieved in only 50% and complications were observed in 38% [6]. Alternatively, ESD can be performed. The R0-resection rate of ESD is higher compared with EMR, but ESD is associated with a high risk of perforation (up to 39%) and requires extensive experience with endoscopic resection techniques [15–17]. Because of the poor results of endoscopic resection in the duodenum, laparoscopy is most commonly used to remove SETs in this location. However, since limited resection is often not an option in SETs, laparoscopic resection in the duodenum usually consists of a (pylorus-preserving) pancreaticoduodenectomy, an operation associated with significant post-surgical morbidity [1, 2].

In conclusion, all methods available for resection of duodenal SETs have limitations. Nevertheless, eFTR could be considered if resection is indicated, since R0-resection and FTR were achieved in 100% of patients. However, the risk of (micro)perforation needs to be lowered by modification of the applied technique or design of the clip. Until this is achieved, duodenal eFTR should not be considered standard clinical practice. If eFTR is considered the only viable option, e.g., in case of significant morbidity but also the presence of comorbidities, the risk of (micro)perforation should warrant hospitalization for observation and prophylactic antibiotics. Fortunately, all complications could be managed conservatively, with only one Grade III complication, thereby comparing favorably to surgery. There are several possible explanations for the high number of complications observed in duodenal eFTR. First and foremost, the relatively thin wall of the duodenum, approximately 1.5 mm on average, is very fragile; when the ‘swords’ turn inwards after puncturing the duodenum, the swords may tear the duodenum which causes a (micro)perforation, especially when the clip and snare make contact during electrosurgical snaring of the lesion. Moreover, electrosurgical snaring may cause deep coagulation damage, which could affect tissue strength. Another possible explanation is that the space between the wider parts of the opposing swords (9 mm) could be too large for the thin duodenum, which allows for the two walls to give way which in turn results in a perforation [18]. After the first microperforation, it was decided to leave more tissue in situ above the clip. Despite this more defensive approach however, delayed bleeding occurred for which, apart from the extensive vasculature of the duodenum, no causative factor was found. Dedicated effort was put in avoiding the clip with the electrosurgical snare; nevertheless, abdominal infection due to perforation occurred.

Reported results of EMR, ESD, and surgical resection for SETs in the stomach are better than those for the duodenum. Bleeding rates after ESD and EMR are comparable (approximately 10%), procedure time for ESD is longer (approximately 60 vs. 15 min for EMR), and the perforation risk of ESD is higher (approximately 4 vs. 1% for EMR) [7, 8]. However, high en bloc resection and R0-resection rates for ESD of gastric SETs situated above the muscularis propria layer are the reason that ESD is considered first-line treatment in this location [7–9, 19, 20]. Performing ESD in gastric SETs originating from the muscularis propria is feasible (R0-resection in 97.1%), but is associated with an increased risk of perforation (12.1%) [21]. Alternatively, laparoscopic resection can be performed, especially in larger SETs originating from the muscularis propria. Limited laparoscopic resection is often performed for SETs in the stomach, which is effective and safe, but is time consuming and requires multiple days of post-surgical admission [3, 4, 22]. In the current study, R0-resection was achieved in all successfully clipped gastric SETs. Furthermore, the median number of in-hospital days after gastric eFTR was just 1 day in the current study, compared with 5 days after ESD in a recent study [22]. The sample size of this study is too small for comparison to ESD with regard to the risk of complication. Nevertheless, eFTR with this new flat-based clip is a promising new technique for gastric SETs < 20 mm situated superficial to the muscularis propria and should be further explored in larger studies. Two gastric lesions originating from the muscularis propria could not be suctioned in the cap. The muscularis propria was less compliant than more superficial layers upon suctioning, which combined with the fact that the two lesions were relatively large (18 and 20 mm) may well have caused the failure to suction the lesions in the cap. Therefore, it is advisable to perform eFTR of gastric lesions located in the muscularis propria of lesions up to 15 mm only. However, larger studies are required to test this hypothesis.

Literature on eFTR in the stomach and duodenum is scarce. Sarker et al. [23] reported on eFTR of three SETs in the duodenum and two in the stomach with a different, curved, OTS clip (OTSC; Ovesco Endoscopy AG, Tübingen, Germany) and reported no adverse events. Although R0-resection was achieved in all five cases, FTR was achieved in only one gastric NET. Three other studies report on their experience with the same type of clip. Fahnrich et al. [24] reported one successful eFTR in the duodenum with R0-resection and two gastric lesions with R0 in both and FTR in one. Schmidt et al. [25] reported on eFTR of two SETs in the duodenum; R0-resection and FTR were achieved in both. Monkemuller et al. [26] reported on successful eFTR of one gastric NET but did not report whether R0 and FTR were achieved. In all studies, no adverse events were reported. Recently, another retrospective study reported on four eFTR procedures of upper gastrointestinal SETs with the flat-based OTS clip [11]. However, placement of the flat-based OTS clip was successful in only one case (duodenal bulb). Two SETs that could not be clipped were located in the duodenal bulb and one was located in the lesser curve of the stomach. In all three cases, the clip failed to deploy. The curved OTSC was used in the three failed cases and in one other case. In all five included upper GI SETs (four located in the duodenum), R0-resection was achieved and no adverse events were reported. It was not specified whether FTR was achieved.

The rate of FTR achieved in the duodenum in these other studies is somewhat low; we were able to accomplish FTR in all SETs in the duodenum and R0-resection of all duodenal and gastric SETs. En bloc resection in all successful cases allows for a reliable judgment of whether R0-resection is achieved, as opposed to piecemeal resection in EMR. Furthermore, resection with this OTS clip was considered fairly easy, while other techniques (e.g., EMR, ESD) require extensive experience in endoscopic resection techniques [16]. Therefore, we believe that the flat-based OTS clip may be a useful addition to the arsenal of the therapeutic endoscopist for gastric SETs < 20 mm in size and located above the muscularis propria. Moreover, if the design of the clip and technique for resection of duodenal lesions < 20 mm are optimized, then this would have important implications for the treatment of duodenal SETs in the foreseeable future.

In conclusion, gastric and duodenal eFTR with this new, flat-based OTS clip is feasible. However, the resection technique should be further optimized before eFTR of duodenal SETs can be performed safely on a larger scale. eFTR in the stomach is effective with a favorable safety profile. High RO- and en bloc resection rates make eFTR with this clip an interesting new device for therapeutic endoscopists.

## Electronic supplementary material

Below is the link to the electronic supplementary material.

Supplemental video 1: Pseudopolyp resection and removal with an electrosurgical snare after Padlock placement. (MP4 213023 KB)

## References

[1] Yang F, Jin C, Du Z, Subedi S, Jiang Y, Li J (2013). Duodenal gastrointestinal stromal tumor: clinicopathological characteristics, surgical outcomes, long term survival and predictors for adverse outcomes. Am J Surg.

[2] Rangelova E, Blomberg J, Ansorge C, Lundell L, Segersvard R, Del Chiaro M (2015). Pancreas-preserving duodenectomy is a safe alternative to high-risk pancreatoduodenectomy for premalignant duodenal lesions. J Gastrointest Surg.

[3] Koh YX, Chok AY, Zheng HL, Tan CS, Chow PK, Wong WK (2013). A systematic review and meta-analysis comparing laparoscopic versus open gastric resections for gastrointestinal stromal tumors of the stomach. Ann Surg Oncol.

[4] Bischof DA, Kim Y, Dodson R, Carolina Jimenez M, Behman R, Cocieru A (2014). Open versus minimally invasive resection of gastric GIST: a multi-institutional analysis of short- and long-term outcomes. Ann Surg Oncol.

[5] Delle Fave G, O’Toole D, Sundin A, Taal B, Ferolla P, Ramage JK (2016). ENETS consensus guidelines update for gastroduodenal neuroendocrine neoplasms. Neuroendocrinology.

[6] Gincul R, Ponchon T, Napoleon B, Scoazec JY, Guillaud O, Saurin JC (2016). Endoscopic treatment of sporadic small duodenal and ampullary neuroendocrine tumors. Endoscopy.

[7] Lian J, Chen S, Zhang Y, Qiu F (2012). A meta-analysis of endoscopic submucosal dissection and EMR for early gastric cancer. Gastrointest Endosc.

[8] Facciorusso A, Antonino M, Di Maso M, Muscatiello N (2014). Endoscopic submucosal dissection vs endoscopic mucosal resection for early gastric cancer: a meta-analysis. World J Gastrointest Endosc.

[9] Pimentel-Nunes P, Dinis-Ribeiro M, Ponchon T, Repici A, Vieth M, De Ceglie A (2015). Endoscopic submucosal dissection: European society of gastrointestinal endoscopy (ESGE) guideline. Endoscopy.

[10] Armellini E, Crino SF, Orsello M, Ballare M, Tari R, Saettone S (2015). Novel endoscopic over-the-scope clip system. World J Gastroenterol.

[11] Al-Bawardy B, Rajan E, Wong Kee Song LM (2016). Over-the-scope clip-assisted endoscopic full-thickness resection of epithelial and subepithelial GI lesions. Gastrointest Endosc.

[12] ESMO/European Sarcoma Network Working Group (2014). Gastrointestinal stromal tumours: ESMO clinical practice guidelines for diagnosis, treatment and follow-up. Ann Oncol.

[13] Dindo D, Demartines N, Clavien PA (2004). Classification of surgical complications: a new proposal with evaluation in a cohort of 6336 patients and results of a survey. Ann Surg.

[14] Clavien PA, Barkun J, de Oliveira ML, Vauthey JN, Dindo D, Schulick RD (2009). The Clavien-Dindo classification of surgical complications: five-year experience. Ann Surg.

[15] Kim TW, Kim GH, Park DY, Ahn S, Lim W, Lee BE (2016). Endoscopic resection for duodenal subepithelial tumors: A single-center experience. Surg Endosc.

[16] Basford P, Bhandari P (2016). Endoscopic resection of sporadic duodenal neuroendocrine tumors: why is this not so easy?. Endoscopy.

[17] Matsumoto S, Yoshida Y (2014). Selection of appropriate endoscopic therapies for duodenal tumors: an open-label study, single-center experience. World J Gastroenterol.

[18] Cronin CG, Delappe E, Lohan DG, Roche C, Murphy JM (2010). Normal small bowel wall characteristics on MR enterography. Eur J Radiol.

[19] Lee JS, Kim GH, Park DY, Yoon JM, Kim TW, Seo JH (2015). Endoscopic submucosal dissection for gastric subepithelial tumors: a single-center experience. Gastroenterol Res Pract.

[20] Libanio D, Pimentel-Nunes P, Dinis-Ribeiro M (2016). Complications of endoscopic resection techniques for upper GI tract lesions. Best Pract Res Clin Gastroenterol.

[21] Ye LP, Zhang Y, Luo DH, Mao XL, Zheng HH, Zhou XB (2016). Safety of endoscopic resection for upper gastrointestinal subepithelial tumors originating from the muscularis propria layer: an analysis of 733 tumors. Am J Gastroenterol.

[22] Soh JS, Kim JK, Lim H, Kang HS, Park JW, Kim SE (2016). Comparison of endoscopic submucosal dissection and surgical resection for treating gastric subepithelial tumours. Scand J Gastroenterol.

[23] Sarker S, Gutierrez JP, Council L, Brazelton JD, Kyanam Kabir Baig KR, Monkemuller K (2014). Over-the-scope clip-assisted method for resection of full-thickness submucosal lesions of the gastrointestinal tract. Endoscopy.

[24] Fahndrich M, Sandmann M (2015). Endoscopic full-thickness resection for gastrointestinal lesions using the over-the-scope clip system: a case series. Endoscopy.

[25] Schmidt A, Meier B, Cahyadi O, Caca K (2015). Duodenal endoscopic full-thickness resection (with video). Gastrointest Endosc.

[26] Monkemuller K, Peter S, Toshniwal J, Popa D, Zabielski M, Stahl RD (2014). Multipurpose use of the ‘bear claw’ (over-the-scope-clip system) to treat endoluminal gastrointestinal disorders. Dig Endosc.

